# On the Common Syringe with a Flexible Tube, as Applicable to the Removal of Opium and Other Poisons from the Stomach

**Published:** 1822-09

**Authors:** F. Bush

**Affiliations:** Surgeon, Frome.


ART. IX.—*On the common Syringe with a flexible Tube, as applicable to the Removal of Opium and other Poisons from the Stomach.* By F. BUSH, Surgeon, Frome.

THE common occurrence of death from opium, either when taken by accident or design, shows that we have hitherto not been acquainted with any certain means of ejecting it from the stomach, or of counteracting its effect. I have constructed an instrument that might, perhaps, properly enough, be called the *gastric exhauster:* it is not meant to supersede the use of emetics and other medicines, though I am of opinion it may, in many cases, render their use less necessary than formerly. The instrument consists of a common syringe, with a flexible tube fitted to it, of length sufficient to allow one end to enter the stomach: it may be formed of leather, or of the same materials as the elastic flexible bougie, (the same syringe should have these tubes of different sizes, to suit the various subjects who might require their use;) the ordinary circumference of it would be from an inch to an inch and a half; the point destined to enter the stomach should be smooth and furnished with holes, like the catheter: these holes should be four, or six, in number, and as large as the tube will admit of. By attention to this part of its construction, even the more solid, as well as the fluid, contents of the stomach will be brought from it, when the piston is made to act. The syringe and the tube should not be permanently fixed, but so constructed that they can be put together and separated instantaneously; and a flexible rod of whalebone should accompany the tube, to give firmness to it in entering the stomach, but should be withdrawn before the syringe is fixed.

I will add a few remarks on the mode of using this instrument. If called to a person who had swallowed opium, I would introduce the tube of the exhauster into the stomach; having half filled the syringe (which should at least be capable of containing a pint,) with warm water, I would fix it to the tube, and, in the most gentle manner, force it into the stomach; then, with the same caution, by drawing back the piston, fill the syringe with the contents of the stomach, and, by separating the syringe from the tube, discharge it. I would then fill the syringe partially as before, and repeat the process above described; and so again and again, till the water was returned unmixed with the poison. The patient might be in the sitting position on the introduction of the tube, but lying on the left side might be a better one, when the injection and exhaustion is to be performed. The introduction of the water would seem a necessary precaution, to prevent ill effects from the action of the exhauster on a comparatively empty stomach.

This operation, if conducted with the least address, might be commenced and finished in three minutes; and all the danger from the retention of the poison in the stomach, which takes place when emetics are relied on, avoided.

These observations were suggested by the following case; which is the only one I have ever witnessed where opium was taken in sufficient quantity to excite the apprehension of danger.

Case.—A gentleman, aged sixty-five years, of sanguine temperament, in a state of intoxication, which had been aided by smoking tobacco, drank an ounce and a half of tincture of opium. It was soon discovered; and the advice of Mr. Yeatman, of this place, being obtained, his efforts were directed to the dislodgment of the poison from the stomach; and for that purpose, as he informed me, he administered large and repeated doses of sulphate of zinc, but without producing vomiting, although the fauces were at the same time irritated to provoke this effect. About thirteen hours after the poison had been taken, I was requested to visit the patient, whose case was then considered of great danger: my visit took place about an hour and a half before death. I cannot speak of the symptoms as they occurred through the early stage of this case. When I first saw the patient, the countenance was bloated, the lips blue, the eyes without expression, great stupor, and a constant falling into sleep, though roused by shaking; the breathing was tolerably free, the pulse at the wrist rather small and at 90; the hands and feet were cold. He had the power to controul the voluntary muscles even a few minutes before death; for at that time he expressed a wish to void urine: a pot was placed for his use, and he did so, to the extent of a pint or more, which was quite natural in appearance. Opium taken into the stomach renders that organ so insensible to the ordinary provocatives to vomiting, that in many cases, although they do ultimately produce that effect, the poison is allowed to remain long enough in the stomach to occasion death.

In this case there did not appear to be congestion on the brain; but the heart appeared to have lost its sensibility, and no longer obeyed its ordinary stimulus, the blood.

*July 23d*, 1822.

